# Supplementary material for: Suppression and facilitation of human neural responses
Source: eLife. 2018 Jan 29;7:e30334. doi: 10.7554/eLife.30334 (PMC5812713; doi:10.7554/eLife.30334)
Supplement: Transparent reporting form [file elife-30334-transrepform.docx]

***eLife’s* transparent reporting form**

We encourage authors to provide detailed information *within their submission* to facilitate the interpretation and replication of experiments. Authors can upload supporting documentation to indicate the use of appropriate reporting guidelines for health-related research (see [EQUATOR Network](http://www.equator-network.org/%20)), life science research (see the [BioSharing Information Resource](https://biosharing.org/)), or the [ARRIVE guidelines](http://www.plosbiology.org/article/info:doi/10.1371/journal.pbio.1000412) for reporting work involving animal research. Where applicable, authors should refer to any relevant reporting standards documents in this form.

If you have any questions, please consult our Journal Policies and/or contact us: [editorial@elifesciences.org](mailto:editorial@elifesciences.org).

**Sample-size estimation**

- You should state whether an appropriate sample size was computed when the study was being designed
- You should state the statistical method of sample size computation and any required assumptions
- If no explicit power analysis was used, you should describe how you decided what sample (replicate) size (number) to use

Please outline where this information can be found within the submission (e.g., sections or figure legends), or explain why this information doesn’t apply to your submission:

Power analyses are described in the text where appropriate (e.g., *Statistics* section of the Methods, legends for Supplemental Figures 4, 5, & 6). Full details are included below.

A power analysis was performed on our initial psychophysical data (reported in Fig 2c). To do so, we computed the probability of type II error, assuming an effect size, sample size, and variance equal to that in this first data set. We further assumed that data were normally distributed, and had equal variance between conditions. This power analysis showed that the primary psychophysical effect of interest (positive size indices for 3% contrast stimuli [spatial summation] vs. negative indices for 98% contrast [spatial summation]) was extremely robust. Indeed, we calculated that based on the size and variability of this effect across subjects (n = 10), the probability of type II error was essentially zero.

Likewise, we calculated the probability of type II error in our fMRI experiment using our initial data set (Fig 3). This showed that for the primary effect of interest (suppression vs. enhancement of the fMRI response at 98 and 3%, respectively, with increasing stimulus size), the probability of type II error was less than 10%, given the effect size and variance observed (with n = 8 subjects for hMT+, and n = 10 for EVC, averaging across stimulus sizes).

The same kind of power analysis was performed for the Lorazepam experiment (Fig 4), which showed that, based on the variance and effect size observed in the first subject (thresholds for lorazepam vs. placebo, averaged across conditions), the likelihood of type II error was less than 1% with a sample size of 15.

Finally, for the correlation between GABA MRS and psychophysical thresholds (Fig 5d), we performed a power analysis assuming a correlation coefficient of *r* = 0.6 (based on pilot data), which showed that 20 subjects would be required for a probability of type II error less than 20%. Our actual sample sizes (n = 21 to 27) allow us to detect correlations of *r* ≥ 0.57 with the same power (type II error less than 20%), assuming a significance threshold of *p* = 0.05.

**Replicates**

- You should report how often each experiment was performed
- You should include a definition of biological versus technical replication
- The data obtained should be provided and sufficient information should be provided to indicate the number of independent biological and/or technical replicates
- If you encountered any outliers, you should describe how these were handled
- Criteria for exclusion/inclusion of data should be clearly stated
- High-throughput sequence data should be uploaded before submission, with a private link for reviewers provided (these are available from both GEO and ArrayExpress)

Please outline where this information can be found within the submission (e.g., sections or figure legends), or explain why this information doesn’t apply to your submission:

This information is included in the relevant Methods sections (e.g., *Participants*, *Psychophysics*), and in the relevant figure legends.

**Statistical reporting**

- Statistical analysis methods should be described and justified
- Raw data should be presented in figures whenever informative to do so (typically when N per group is less than 10)
- For each experiment, you should identify the statistical tests used, exact values of N, definitions of center, methods of multiple test correction, and dispersion and precision measures (e.g., mean, median, SD, SEM, confidence intervals; and, for the major substantive results, a measure of effect size (e.g., Pearson's r, Cohen's d)
- Report exact p-values wherever possible alongside the summary statistics and 95% confidence intervals. These should be reported for all key questions and not only when the p-value is less than 0.05.

Please outline where this information can be found within the submission (e.g., sections or figure legends), or explain why this information doesn’t apply to your submission:

This information is included both in the Methods section (e.g., *Statistics*), and in the relevant figure legends.

(For large datasets, or papers with a very large number of statistical tests, you may upload a single table file with tests, Ns, etc., with reference to sections in the manuscript.)

**Group allocation**

- Indicate how samples were allocated into experimental groups (in the case of clinical studies, please specify allocation to treatment method); if randomization was used, please also state if restricted randomization was applied
- Indicate if masking was used during group allocation, data collection and/or data analysis

Please outline where this information can be found within the submission (e.g., sections or figure legends), or explain why this information doesn’t apply to your submission:

This is not relevant to our study. The lorazepam experiment was performed within-subjects, in a double-blinded fashion, as noted in the Methods.

**Additional data files (“source data”)**

- We encourage you to upload relevant additional data files, such as numerical data that are represented as a graph in a figure, or as a summary table
- Where provided, these should be in the most useful format, and they can be uploaded as “Source data” files linked to a main figure or table
- Include model definition files including the full list of parameters used
- Include code used for data analysis (e.g., R, MatLab)
- Avoid stating that data files are “available upon request”

Please indicate the figures or tables for which source data files have been provided:

We provide a reference for our modeling work (Reynolds & Heeger, 2009); the authors of that paper provide an implementation of their model in Matlab, which we adapted for the current study.
